# The double-edged sword of scapegoration: how anti-immigrant hostility harms both immigrants and host societies

**DOI:** 10.3389/fpsyg.2026.1809730

**Published:** 2026-08-20

**Authors:** Changiz Mohiyeddini

**Affiliations:** 1 Michael Garron Hospital, Toronto East Health Network, Toronto, ON, Canada; 2 Department of Family and Community Medicine, Temerty Faculty of Medicine, University of Toronto, Toronto, ON, Canada

**Keywords:** crimmigration, dehumanization, immigration, moral disengagement, psychological trauma, scapegoration, social determinants of health, social identity

## Abstract

Immigration is among the most consequential social phenomena of the 21st century, yet the psychological dimensions of anti-immigrant hostility—particularly its bidirectional harms—remain insufficiently theorized. This paper introduces and defines the concept of scapegoration, a portmanteau of scapegoating and immigration, to describe the systematic attribution of structural societal failures to immigrant populations as a means of deflecting responsibility from institutional and political actors. Unlike xenophobia, which denotes generalized fear or hostility toward foreigners, scapegoration captures a specific politico-psychological process in which immigrants are instrumentally constructed as causal agents of pre-existing social problems. Building upon Albert Memmi's (1957) framework in The Colonizer and the Colonized and integrating Integrated Threat Theory, Social Identity Theory, moral disengagement theory, and the concept of crimmigration, this paper argues that the criminalization and scapegoating of immigrants inflict psychological harm on both migrants and host populations. The analysis operates across individual, group, institutional, and societal levels to demonstrate how scapegoration functions as a self-perpetuating system of exclusion and dehumanization. Drawing on clinical psychology, sociology, political theory, and critical migration studies, the paper examines how host societies develop moral hypocrisy, epistemic distortion (the systematic misrepresentation or misattribution of social reality through politically reinforced narratives), and cultural paranoia (persistent and exaggerated beliefs that cultural identity is under existential threat from immigrant populations), while immigrants experience trauma, exploitation, intergenerational harm, and identity disruption. The paper concludes with an integrated multilevel framework for counter-scapegoration interventions encompassing policy reform, narrative change, and structural inclusion.

## Introduction

The 21st century has witnessed an unprecedented wave of global human movement. The International Organization for Migration (IOM, 2024) estimates over 280 million international migrants, driven by conflict, climate change, economic collapse, and political instability. While sociopolitical analyses of immigration abound, the psychological dimensions remain underexplored in policy discourse. In particular, although extensive research has documented the mental health consequences of migration for immigrants themselves (Berry, 1997; Schwartz et al., 2015), comparatively little systematic attention has been given to the psychological processes by which host societies construct immigrants as threats—and to the reciprocal damage these constructions inflict upon the host populations that deploy them.

Immigration refers to the international movement of individuals who relocate to a destination country where they are not native residents or citizens, typically with the intention of settling permanently (United Nations, 2026; IOM, 2019). Historically, human migration has taken diverse forms, from early population movements during the Upper Paleolithic to modern-day voluntary and involuntary migration, including forced displacement due to conflict, persecution, or environmental crises (IOM, 2019). Contemporary migration patterns are shaped by geopolitical, economic, and social factors, with distinctions between regulated and unauthorized immigration carrying significant legal and psychosocial consequences (Massey et al., 2016). Classic accounts of immigrant incorporation further emphasise that integration typically unfolds across generations through labour market participation, education, and social ties, rather than as a single-generation event (Alba and Nee, 2003; Portes and Rumbaut, 2014). Economically, research indicates that migration can yield benefits for both receiving and sending nations, though findings on the relationship between immigration and crime remain mixed (Borjas, 2014; Bell and Machin, 2013). Crucially, meta-analytic evidence consistently demonstrates that immigration does not increase—and may in fact reduce—violent crime rates (Light and Miller, 2018; Ousey and Kubrin, 2022), a finding that stands in stark contrast to public perception.

Despite these dynamics, discrimination based on nationality persists as a legally permissible practice in many countries, with extensive evidence documenting bias against foreign-born individuals across multiple domains, including criminal justice, employment, housing, healthcare, and politics (Pager and Shepherd, 2008; Quillian et al., 2019). Such discrimination is frequently undergirded by xenophobia—defined as the fear or hatred of that which is perceived as foreign or strange (Peterie and Neil, 2020). Xenophobia operates at the level of affect and disposition, generating generalized hostility toward immigrants as a category. However, as Tummala-Narra (2020) has argued from a psychoanalytic perspective, the fear of immigrants is not merely a reaction to perceived external threat but reflects deeper anxieties about identity coherence, cultural continuity, and the stability of the self in the face of difference. Lee (2020) has further demonstrated the historical recurrence of xenophobic sentiment in American political life, showing that “America First” nativism is not a contemporary aberration but a deeply rooted political tradition that resurfaces during periods of economic and cultural anxiety. These insights are essential for understanding the broader affective landscape within which scapegoration operates, but they also reveal why xenophobia alone is an insufficient concept for capturing the phenomenon this paper addresses.

However, in many contemporary societies, immigration has increasingly become framed through narratives emphasizing threat, risk, and social disruption.

This transformation is not merely a matter of xenophobic sentiment or generalized prejudice. It reflects a more specific and instrumentalized process that I term scapegoration—a portmanteau of *scapegoating* and *immigration*. Scapegoration is defined as the systematic, politically instrumentalized attribution of structural societal failures—economic precarity, public safety anxieties, institutional dysfunction—to immigrant populations, deployed as a mechanism of collective psychological defense and political mobilization. Scapegoration differs from xenophobia in three critical respects. First, whereas xenophobia denotes a dispositional affective state (fear or hostility toward foreigners), scapegoration describes a dynamic process with identifiable stages: threat construction, causal misattribution, political amplification, and policy crystallization. Second, scapegoration is instrumentally strategic rather than merely reactive; it is cultivated by political actors, media systems, and institutional practices for identifiable purposes. Third, scapegoration produces bidirectional harm—damaging not only its targets but also the moral, epistemic, and democratic capacities of the societies that deploy it.

## Distinguishing scapegoration from related constructs

Scapegoration shares conceptual territory with several established constructs, including scapegoating, anti-immigrant prejudice, dehumanization, and othering. However, it is not reducible to any of these concepts.

Scapegoating refers broadly to the attribution of blame for social, economic, or political problems to a vulnerable outgroup (Allport, 1954). Scapegoration may be understood as a specific subtype of scapegoating in which immigrant populations become the preferred targets of blame attribution. Unlike generic scapegoating, scapegoration focuses specifically on migration and immigration-related populations and examines the institutional processes through which such blame becomes embedded in public policy.

Anti-immigrant prejudice refers to negative attitudes toward immigrants and immigration. Prejudice is primarily an attitudinal construct. Scapegoration, by contrast, describes a dynamic socio-political process that extends beyond attitudes to include threat construction, causal misattribution, political mobilization, policy formation, and institutional reinforcement.

Dehumanization involves denying the humanity of another group (Haslam, 2006). While dehumanization frequently occurs within scapegoration, it represents one psychological mechanism through which scapegoration operates rather than the broader process itself.

Similarly, othering refers to the symbolic construction of social boundaries separating “us” from “them” (Said, 1978). Othering contributes to scapegoration by creating categories of exclusion, but it does not necessarily involve blame attribution or the systematic transfer of responsibility for societal problems.

Thus, scapegoration is best understood as a multilevel process that incorporates elements of prejudice, othering, and dehumanization while adding a distinct mechanism of politically instrumentalized blame attribution directed toward immigrant populations.

Albert Memmi’s *The Colonizer and the Colonized* (Memmi, 1957) remains one of the most incisive psychological analyses of oppression, revealing how systemic domination distorts the identities of both oppressor and oppressed. Though Memmi focused on colonial regimes, his framework applies strikingly to modern immigration systems where host countries—particularly Western nations with colonial histories—replicate colonial logic by criminalizing, excluding, and scapegoating migrants. This paper argues that the psychological mechanisms Memmi identified—moral hypocrisy among dominators, internalized inferiority among the subjugated, and the systemic erosion of communal well-being—manifest today in immigration policies framed as “security measures” or “economic protections.”

Building on Memmi’s insight, I argue that scapegoration does not merely harm immigrants; it degrades host societies by normalizing dehumanization, wasting economic resources, and fostering cultural stagnation. Clinical research shows that both groups suffer: immigrants endure trauma akin to colonized subjects (Roth et al., 2018), while host populations exhibit rising xenophobia linked to authoritarianism (Drotbohm and Hasselberg, 2015). Immigration debates constitute a psychological crisis in three interrelated senses: they activate mass-level threat responses that override rational evaluation of evidence (Stephan and Stephan, 2000); they erode the moral-cognitive capacities required for democratic deliberation through processes of moral disengagement (Bandura, 1999); and they produce measurable mental health consequences for immigrant populations whose wellbeing deteriorates under hostile policy environments (Garcini et al., 2017; Hainmueller et al., 2017). The crisis, in other words, is not metaphorical—it is clinical, civic, and epistemic simultaneously. By applying Memmi’s dialectic to contemporary migration, this paper reframes immigration debates as a psychological crisis demanding systemic intervention.

To understand how scapegoration operates at both individual and collective levels, the analysis that follows is organized across four interdependent levels: (a) individual psychological mechanisms, including projection, threat perception, and moral disengagement; (b) group-level dynamics, encompassing social identity processes, collective trauma, and in-group solidarity; (c) institutional processes, including the convergence of criminal and immigration law (“crimmigration”), the structural production of illegality, and the role of organizations in embedding exclusion; and (d) societal-level phenomena, including the erosion of democratic norms, epistemic distortion, and intergenerational trauma transmission. This multilevel architecture addresses the need for theoretical coherence across scales of analysis and clarifies how scapegoration differs from related but distinct constructs such as xenophobia, systemic racism, and structural violence.

### The psychology of scapegoration: individual and group-level mechanisms

Scapegoration operates by attributing economic, cultural, or security threats to immigrants, particularly in times of political instability or economic anxiety. Psychological research shows that scapegoating serves as a defensive projection mechanism, allowing majority groups to externalize systemic failures onto marginalized out-groups (Allport, 1954; Glick, 2005). Immigrants, especially those who are undocumented or racialized, become symbolic targets for broader societal frustrations. At the individual psychological level, this process involves a specific sequence: the experience of threat or frustration, the inability or unwillingness to identify the actual structural sources of that threat (e.g., deindustrialization, wage stagnation, policy failures), and the displacement of aggressive impulses onto a socially available and politically sanctioned target. Moreno et al. (2025) have recently demonstrated how the intersection of immigration-related racism and xenophobia shapes undocumented immigrants’ experiences of liminality—a perpetual state of being betwixt and between that is itself a product of scapegorative social structures.

This psychological process is often reinforced by media narratives and populist political discourse that depict immigrants as burdens or threats (Esses et al., 2013). The result is a climate of suspicion and exclusion, one that not only causes measurable harm to immigrants but also destabilizes host societies by fostering authoritarianism and institutional distrust. Matthes and Schmuck (2017) demonstrated experimentally that exposure to right-wing populist anti-immigrant advertising strengthens both implicit and explicit negative attitudes toward immigrants, with the effects mediated by perceived threat—findings that underscore the constructed rather than spontaneous nature of scapegorative sentiment.

For immigrants, scapegoration is linked to chronic stress, social invisibility, restricted access to essential services, and legal precarity (Castañeda et al., 2015; Hacker et al., 2015). The concept of immigration as a social determinant of health, as theorized by Castañeda et al. (2015), provides a critical conceptual bridge between scapegoration and its health consequences. Social determinants of health refer to the conditions in which people are born, grow, live, work, and age—conditions shaped by the distribution of money, power, and resources (WHO, 2008). When immigration status itself becomes a determinant, it does so not because of any intrinsic characteristic of migrants but because of the structural barriers, legal exclusions, and hostile environments that scapegorative policies create. Hacker et al. (2015) documented how undocumented immigrants face compounding barriers to healthcare access—including fear of deportation, lack of insurance eligibility, language barriers, and provider discrimination—that function as downstream consequences of scapegorative policy architectures. These barriers do not merely limit healthcare utilization; they generate a cascade of avoidance behaviors affecting engagement with education, law enforcement, and civic institutions, thereby producing the very social marginality that scapegorative rhetoric then attributes to immigrant cultures themselves. These social determinants translate into health disparities—elevated rates of anxiety, depression, and PTSD are well-documented among immigrants subjected to hostile environments (Garcini et al., 2017).

### Psychological and biological pathways linking scapegoration and health

Several interrelated psychological mechanisms may explain how scapegoration influences health outcomes. First, persistent exposure to discrimination, uncertainty, and social exclusion may activate chronic stress responses, resulting in prolonged activation of physiological stress systems, including the hypothalamic–pituitary–adrenal axis (McEwen, 1998). Second, anticipatory vigilance regarding discrimination, detention, or deportation may increase anxiety and reduce perceptions of safety and control. Third, social exclusion may diminish social support and belongingness, both of which are well-established protective factors for psychological and physical health (Cohen et al., 2007). Finally, repeated experiences of stigmatization may contribute to internalized shame, identity conflict, and reduced help-seeking behavior. These mechanisms are consistent with minority stress frameworks, which propose that chronic exposure to stigma and discrimination creates cumulative psychological and physiological burdens (Meyer, 2003). Together, these pathways provide a plausible health psychological account of how scapegoration may influence both mental and physical wellbeing.

### The psychology of host societies: what drives scapegoration?

Host societies that embrace scapegoration risk undermining their own democratic norms. Research shows that exclusionary immigration policy contributes to political polarization, weakens public trust, and promotes carceral expansion under the guise of border security (Wodak, 2015; García Hernández, 2019).

While the experiences and psychological toll on migrants have been extensively documented, the psyche of the host society—particularly its tendency to turn vulnerable groups into symbols of national anxiety—remains underexplored. This essay investigates the psychological roots, cultural facilitators, and systemic reinforcers of scapegoration, drawing on recent empirical studies and theoretical frameworks in social, political, and cultural psychology.

Scapegoration occurs when host societies systematically associate immigrants with criminality, moral decline, or existential threat, regardless of actual data or empirical risk. In psychological terms, this is not simply a matter of prejudice or stereotyping, but a projection process that fulfills deep-seated emotional and social needs. According to Allport’s (1954) seminal work on prejudice, scapegoating arises when dominant groups, under stress or threat, transfer their aggression onto weaker, socially available outgroups. Allport’s framework, though foundational, requires updating in light of contemporary developments. Whereas Allport conceptualized prejudice primarily as an individual cognitive phenomenon, scapegoration operates across multiple levels simultaneously—individual, group, institutional, and societal—and is actively cultivated rather than merely emerging from ignorance or cognitive limitation.

Recent developments in Integrated Threat Theory (Stephan and Stephan, 2000) provide a compelling explanation for the surge in scapegoration during periods of societal instability. This theory delineates two types of threat that drive negative intergroup attitudes: realistic threats, concerning economic or physical security (e.g., jobs, crime, terrorism), and symbolic threats, involving identity, values, and cultural norms. Immigration—particularly when framed through a lens of “invasion” or “flooding” by political elites or media—activates both threat types simultaneously. Integrated Threat Theory thus provides the individual-level mechanism through which scapegoration operates: it explains why people are psychologically receptive to scapegorative narratives, even when those narratives contradict available evidence. The theory predicts that scapegoration will intensify precisely when both realistic and symbolic threats are perceived simultaneously—a condition that populist political movements deliberately engineer through strategic communication (Mudde and Rovira Kaltwasser, 2017).

Crucially, the emotional undercurrent of scapegoration is fear—often irrational and out of proportion to reality. Empirical studies have consistently shown that public perceptions of immigrant criminality vastly overestimate actual crime statistics. For instance, Light and Miller (2018), analyzing data from Texas, found that undocumented immigrants committed crimes at substantially lower rates than native-born citizens. Despite this, political rhetoric and sensationalist media persist in linking immigration with violence and lawlessness, thereby constructing a social reality rooted more in psychological need than factual evidence.

Social Identity Theory (Tajfel and Turner, 1979) further elucidates the mechanics behind this distortion. When national identity is perceived as under siege, group boundaries harden, and loyalty to the in-group becomes heightened. Immigrants are positioned as outsiders who threaten the coherence and purity of the national self-image. In such moments, any crime committed by an immigrant becomes a metonym for the entire group—amplifying perceived threat and justifying exclusion. Social Identity Theory thus operates at the group level of analysis, explaining how individual threat perceptions aggregate into collective identity-protective responses. This transition from individual psychology to group-level dynamics is where scapegoration acquires its political potency: individual anxieties are transformed into collective narratives of endangerment that license discriminatory policy.

Yet, scapegoration does not emerge in a vacuum. It is actively cultivated by political actors, media framing, and policy discourse. In an era of rising right-wing populism, particularly across Europe and North America, immigrant scapegoating has become a strategic political tool. Research by Mudde (2019) and Inglehart and Norris (2016) shows that right-wing parties capitalize on cultural backlash—resentment over immigration, globalization, and multiculturalism—to mobilize support. This mobilization is not merely rhetorical; it reshapes public policy and institutional behavior. For instance, De Genova (2018) illustrates how border regimes and immigration enforcement construct “illegality” not as a neutral legal category but as a racialized moral judgement—creating what he calls a “legal production of migrant criminality.” Fassin (2011) has extended this analysis through his concept of “humanitarian reason,” demonstrating how even ostensibly compassionate immigration policies can reproduce the logic of exclusion by positioning immigrants as objects of pity rather than subjects of rights—a dynamic that sustains the structural conditions of scapegoration even within liberal welfare states.

The psychological impact on host societies is paradoxically corrosive. While scapegoration appears to offer cohesion—uniting the in-group against a common enemy—it ultimately fosters cynicism, moral disengagement, and democratic decline. Bandura’s (1999) theory of moral disengagement describes how people justify harmful behavior through euphemisms, dehumanization, and diffusion of responsibility. These mechanisms are rampant in political discourse on immigration, where terms like “illegals,” “infiltrators,” or “security risks” strip immigrants of their humanity. As moral concern narrows to the in-group, empathy for the out-group erodes—a process exacerbated by media narratives that decontextualize immigrant lives.

Moreover, research has shown that attitudes toward immigrants are shaped not solely by personal experience, but significantly by national narratives and media framing. A meta-analysis by Esses et al. (2013) concluded that media portrayals that emphasize threat and criminality lead to increased support for restrictive immigration policies and decreased willingness to help refugees. The power of media to shape perceived threat—regardless of objective data—reveals how scapegoration is not only emotional but epistemological: it alters what is seen as truth.

Another critical dimension of scapegoration is the role of collective trauma in shaping group psychology. Nations with unresolved historical trauma—whether from war, colonialism, or economic collapse—often seek scapegoats to explain present instability. Bar-Tal et al. (2007) argue that collective memory and victimhood can drive societies into siege mentalities, where the world is seen as hostile and only in-group solidarity ensures survival. In such climates, immigration is not just policy—it is perceived as existential threat. Thus, scapegoration serves a defensive psychological function: it externalizes pain and locates it in the other.

The consequences are far-reaching. Scapegoration undermines democratic norms by eroding equal protection under law, normalizing xenophobia, and enabling authoritarian governance. It also damages civic trust. When laws are enforced unevenly—harshly on immigrants and leniently on others—belief in institutional fairness declines. A society that criminalizes humanitarian acts, such as providing food or shelter to undocumented migrants, may undermine widely shared ethical principles and democratic norms.

### Institutional mechanisms of scapegoration: crimmigration, structural violence, and necropolitics

At the institutional level, scapegoration operates through what Stumpf (2006) has termed “crimmigration”—the convergence of criminal law and immigration enforcement that transforms administrative violations into criminal categories, constructing the very “immigrant criminality” that public discourse invokes to justify further restriction. This institutional mechanism is critical to understanding how scapegoration differs from individual-level xenophobia: whereas xenophobia resides in attitudes and affects, crimmigration embeds scapegorative logic within the routine operations of legal institutions, producing durable structural consequences that persist regardless of any given individual’s prejudice. García Hernández (2019) describes the resulting “migration-to-prison pipeline” as a system in which the treatment of migration becomes increasingly punitive, feeding into a broader carceral logic that harms society at large.

The concept of structural violence, as developed by Galtung (1969) and applied to migration by Willen (2012), provides a further analytical lens. Structural violence refers to harm that is embedded in social structures and manifests as unequal power relations, systematic exclusion from resources, and the denial of fundamental capabilities. Scapegoration is both a product and a producer of structural violence: it emerges from structural inequalities that generate the socioeconomic anxieties displaced onto immigrants, and it produces new forms of structural exclusion through the policies it legitimates. Viruell-Fuentes et al. (2012) have demonstrated how institutional processes—in healthcare, education, and housing—systematically disadvantage immigrant populations while maintaining a veneer of procedural neutrality, producing disparities that are then attributed to deficiencies within immigrant communities rather than to the structural barriers that generated them.

At the most extreme end of this continuum lies what Mbembe (2003) has termed “necropolitics”—the sovereign power to dictate who may live and who is effectively consigned to social death. While scapegoration does not always reach the threshold of necropolitical violence, the logic of disposability that it cultivates creates the conditions under which necropolitical outcomes become possible: the detention of asylum seekers under inhumane conditions, the separation of families, the deportation of individuals to zones of active conflict. Scapegoration thus occupies a position on a continuum that extends from everyday institutional exclusion to extreme state violence, and its theoretical significance lies precisely in illuminating the psychological and political mechanisms that enable societies to move along this continuum without recognizing that they are doing so.

### Counter-scapegoration: interventions and possibilities

Yet, the psychological mechanisms driving scapegoration are not immutable. Research in contact theory (Pettigrew and Tropp, 2006) shows that structured, positive intergroup contact can reduce prejudice and perceived threat. Moreover, interventions that humanize migrants—through storytelling, perspective-taking, or shared community initiatives—can interrupt the dehumanization process. Bruneau and Saxe (2012) found that empathy-based interventions reduced intergroup hostility more effectively when they emphasized common suffering and moral equivalence, rather than statistical arguments or appeals to logic.

Furthermore, critical media literacy can act as a buffer against scapegoration. When individuals are taught to recognize framing devices, question sources, and seek diverse perspectives, their susceptibility to fear-based narratives declines. Matthes and Schmuck (2017) provide experimental evidence that formal education moderates the attitudinal effects of anti-immigrant populist messaging, suggesting that scapegoration’s influence is contingent on the epistemic capacities of the populations it targets. Educational efforts that highlight the contributions of immigrants to society—not merely economically, but culturally and morally—can help reframe the immigrant not as a threat, but as a neighbor, worker, and citizen-in-the-making.

At the policy level, recognizing scapegoration as a psychological phenomenon compels a shift in how immigration discourse is structured. Governments and civil society organizations must go beyond economic arguments and appeal to values of empathy, justice, and shared humanity. Legal language should reflect this shift: replacing terms like “illegal alien” with “undocumented migrant” is not political correctness but psychological precision. It affirms that people cannot be illegal, only actions can.

### Empirical evidence and broader implications

Empirical studies support the claim that scapegoration has real-world consequences. For instance, Arizona’s SB 1070 law—an aggressive anti-immigration statute—was associated with a decline in healthcare utilization among Latino communities, driven by fear rather than legal status (Rhodes et al., 2015). Philbin et al. (2018) demonstrated that state-level immigration enforcement policies function as independent drivers of Latino health disparities, operating through mechanisms of chronic stress, fear, and institutional avoidance that affect documented and undocumented immigrants alike. Hainmueller et al. (2017) provided quasi-experimental evidence from the DACA program showing that protective immigration policies for undocumented mothers yielded measurable improvements in their children’s mental health, offering causal evidence that scapegoration’s harms cascade across generations and—crucially—that policy interventions can interrupt this transmission.

Contrary to scapegorative narratives, immigration has been shown to enhance innovation, productivity, and economic dynamism when supported by inclusive policies (Ottaviano and Peri, 2006). By contrast, policies shaped by scapegoration erode civic pluralism and misdirect resources toward punitive enforcement rather than structural reform. It should be acknowledged that the empirical literature is not uniformly supportive of positive immigration effects in all contexts. Borjas (2014) has documented wage depression among low-skilled native workers in specific labor market segments, and legitimate policy debates exist regarding the pace and scale of immigration.

It is also important to distinguish scapegoration from legitimate public policy concerns regarding immigration management. Governments must address issues related to border administration, labor market integration, public services, and national security. Recognizing the existence of such challenges does not in itself constitute scapegoration. Rather, scapegoration occurs when complex structural problems are disproportionately attributed to immigrant populations despite insufficient empirical support for such attribution. This distinction is essential to prevent the concept from becoming a rhetorical label applied to all forms of immigration restriction or criticism. The argument advanced here is not that immigration carries no costs or challenges, but that scapegoration systematically distorts the evaluation of these costs by attributing structural problems to immigrant populations and thereby foreclosing the policy responses—investment in education, infrastructure, labor market regulation—that would actually address them.

Sociologist César Cuauhtémoc García Hernández (2019) describes this dynamic as the “migration-to-prison pipeline,” whereby the treatment of migration becomes increasingly punitive, feeding into a broader carceral logic that harms society at large—not just immigrants.

### The impact of scapegoration on immigration and future civil society

Scapegoration—the systemic scapegoating of immigrants—remains one of the most destructive dynamics in contemporary societies, with consequences for both migrants and host populations. By attributing economic, cultural, or security anxieties to immigrant groups, scapegoration operates as a defensive projection mechanism, a process identified by Allport (1954) in his seminal analysis of prejudice. This mechanism remains deeply relevant today, even as its manifestations have evolved in the context of globalization, populism, and mass migration.

For immigrants, scapegoration translates into exclusion, precarity, and heightened psychological stress. Studies have consistently found that hostile policy environments contribute to elevated rates of anxiety, depression, and PTSD among migrants (Garcini et al., 2017; Hainmueller et al., 2017). These harms extend beyond individual health: legal precarity drives avoidance of healthcare and civic institutions, producing systemic disparities (Philbin et al., 2018). Such dynamics mirror Memmi’s (1957) observation that oppression corrodes both the oppressed and the oppressor.

For host societies, the impact is equally corrosive. Although research demonstrates that immigrants commit fewer crimes than native-born citizens (Light and Miller, 2018; Ousey and Kubrin, 2022), political and media narratives continue to amplify associations between immigration and criminality. This disconnect between evidence and perception may contribute to authoritarian attitudes and declining democratic trust (Norris and Inglehart, 2019). Beyond these political consequences, scapegoration may also reshape the epistemic environment of host societies by encouraging simplified explanations for complex structural problems. As Fricker (2007) argues, persistent distortions in collective understanding can generate forms of hermeneutical injustice in which societies lack adequate interpretive resources to understand the experiences of marginalized groups. Similarly, Wodak (2015) has shown how populist discourses construct perceived threats that redirect attention away from structural sources of insecurity. As Bandura (1999) theorized, processes of moral disengagement—including dehumanization and euphemistic labeling—further normalize the exclusion of immigrants while eroding empathy and civic responsibility.

At the civic level, scapegoration destabilizes democratic pluralism. When immigrants are cast as existential threats, host societies grow tolerant of rights restrictions and punitive governance, what García Hernández (2019) terms the “migration-to-prison pipeline.” This trend erodes institutional trust and weakens civil society, as laws disproportionately targeting migrants signal uneven protections of justice.

Yet, these mechanisms are not immutable. Contact theory (Pettigrew and Tropp, 2006) and experimental work on the moderators of populist media effects (Matthes and Schmuck, 2017) suggest strategies to mitigate scapegoration by fostering empathy and reducing susceptibility to fear-based narratives. Inclusive policies that emphasize participation and recognition of immigrant contributions bolster not only immigrant well-being but also civic cohesion (Hainmueller and Hopkins, 2014).

### The shared damage: a toxic symbiosis

Memmi (1957) concluded that colonialism harmed all parties, creating a “shared sickness” (p. 151). Scapegoration systems yield similar outcomes. Host societies become less democratic as xenophobia justifies eroding rights (e.g., ICE surveilling citizens; Stumpf, 2006). Immigrants, meanwhile, are pushed into informality, avoiding hospitals or police even when victimized (Philbin et al., 2018). The result is a lose-lose dynamic: nations waste resources on enforcement while migrants’ potential is squandered (e.g., engineers working as day laborers; Mattoo et al., 2008).

### Intergenerational transmission and epistemic consequences

A dimension of scapegoration that warrants particular attention is its intergenerational character. The psychological injuries inflicted by anti-immigrant hostility do not remain confined to the generation that directly experiences them. Research on historical trauma transmission—originally developed in studies of Holocaust survivors and Indigenous populations (Danieli, 1998; Brave Heart and DeBruyn, 1998)—demonstrates that the effects of collective persecution propagate through family systems, shaping attachment patterns, identity formation, and emotional regulation in subsequent generations. Children of immigrants who have been subjected to scapegorative environments internalize a paradoxical dual message: they are simultaneously expected to assimilate into a society that has signaled their fundamental unworthiness. Suárez-Orozco and Suárez-Orozco (2001) documented how this “social mirroring” process leads second-generation youth to develop fragmented identities, oscillating between hypervigilant accommodation and defensive withdrawal.

Furthermore, scapegoration exerts a corrosive effect on the epistemic environment of host societies. When political discourse consistently attributes complex socioeconomic problems to immigrant populations, it forecloses the possibility of accurate causal reasoning about structural inequality, deindustrialization, wage stagnation, and failures of governance. This epistemic distortion creates what philosopher Fricker (2007) terms “hermeneutical injustice”: a condition in which the conceptual resources available to a society are systematically inadequate for understanding the experiences of marginalized groups. Under scapegoration, host populations lose the capacity to diagnose the actual sources of their own distress, while immigrants are denied the interpretive frameworks that would render their suffering intelligible to others. The result is a society that is both morally impoverished and intellectually diminished—unable to solve its own problems precisely because it has invested so heavily in misidentifying their causes.

## Conclusion and future directions

Scapegoration captures a dangerous societal pattern: the misattribution of blame onto immigrant populations for complex social and economic problems. This process exacts a psychological and structural toll on both the scapegoated and the scapegoaters.

Addressing it requires not only empirical clarity but sustained commitment to policies grounded in evidence, empathy, and democratic integrity.

In sum, scapegoration inflicts a dual harm: it traumatizes immigrants while corroding the civic and moral fabric of host societies.

### Theoretical scope and causal inference

The present paper is primarily conceptual and integrative rather than causal in design. Although the framework draws extensively on empirical literature, many of the relationships proposed here should be interpreted as theoretically informed hypotheses rather than definitive causal conclusions. Future longitudinal, experimental, and cross-national studies are needed to evaluate the specific mechanisms and pathways proposed within the scapegoration framework.

Addressing this phenomenon requires a deliberate reorientation of immigration discourse toward evidence, empathy, and democratic values. Without such efforts, the future of civil society risks being shaped less by inclusion and solidarity than by fear and exclusion.

Future research should pursue several complementary directions. First, longitudinal studies tracking the psychological trajectories of immigrant communities across generations under varying policy regimes would strengthen the causal claims advanced here, moving beyond cross-sectional designs that cannot disentangle the effects of scapegoration from preexisting vulnerabilities. Second, experimental and quasi-experimental work examining the efficacy of counter-narrative interventions—including perspective-giving paradigms (Bruneau and Saxe, 2012), structured intergroup contact, and critical media literacy programs—is essential for translating theoretical insight into actionable policy. Third, comparative analyses across national contexts would illuminate how variations in institutional design, colonial history, and democratic culture modulate the intensity and consequences of scapegoration.

## Data Availability

The original contributions presented in the study are included in the article/supplementary material, further inquiries can be directed to the corresponding author.
